# Streptococcal collagen-like surface protein 1 promotes adhesion to the respiratory epithelial cell

**DOI:** 10.1186/1471-2180-10-320

**Published:** 2010-12-15

**Authors:** Shih-Ming Chen, Yau-Sheng Tsai, Chin-Ming Wu, Shuen-Kuei Liao, Ling-Chia Wu, Cherng-Shyang Chang, Ya-Hui Liu, Pei-Jane Tsai

**Affiliations:** 1 Graduate Institute of Clinical Medical Sciences, Chang Gung University, Taoyuan, Taiwan ROC; 2 Institute of Clinical Medicine, College of Medicine, National Cheng Kung University, Tainan, Taiwan ROC; 3 Department of Cell Biology and Anatomy, College of Medicine, National Cheng Kung University, Tainan, Taiwan ROC; 4 Department of Medical Laboratory Science and Biotechnology, College of Medicine, National Cheng Kung University, Tainan, Taiwan ROC; 5 Institute of Basic Medical Sciences, College of Medicine, National Cheng Kung University, Tainan, Taiwan ROC

## Abstract

**Background:**

Collagen-like surface proteins Scl1 and Scl2 on *Streptococcus pyogenes *contain contiguous Gly-X-X triplet amino acid motifs, the characteristic structure of human collagen. Although the potential role of Scl1 in adhesion has been studied, the conclusions may be affected by the use of different *S. pyogenes *strains and their carriages of various adhesins. To explore the *bona fide *nature of Scl1 in adherence to human epithelial cells without the potential interference of other streptococcal surface factors, we constructed a *scl1 *isogenic mutant from the Scl2-defective *S. pyogenes *strain and a Scl1-expressed *Escherichia coli*.

**Results:**

Loss of Scl1 in a Scl2-defective *S. pyogenes *strain dramatically decreased the adhesion of bacteria to HEp-2 human epithelial cells. Expression of Scl1 on the surface of the heterologous bacteria *E. coli *significantly increased adhesion to HEp-2. The increase in adhesion was nullified when Scl1-expressed *E. coli *was pre-incubated with proteases or antibodies against recombinant Scl1 (rScl1) protein. Treatment of HEp-2 cells with rScl protein or pronase drastically reduced the binding capability of Scl1-expressed *E. coli*. These findings suggest that the adhesion is mediated through Scl1 on bacterial surface and protein receptor(s) on epithelial cells. Further blocking of potential integrins revealed significant contributions of α2 and β1 integrins in Scl1-mediated binding to epithelial cells.

**Conclusions:**

Together, these results underscore the importance of Scl1 in the virulence of *S. pyogenes *and implicate Scl1 as an adhesin during pathogenesis of streptococcal infection.

## Background

*Streptococcus pyogenes *causes heterogeneous disease types, including pharyngitis, cellulitis, and bacteremia [1]. The pathogenesis of *S. pyogenes *infection involves an intriguing host-pathogen interplay in which the biological activity of several bacterial virulence products are modulated by host factors [2]. The details of the molecular interaction between the bacterium and the host, as well as their influences on the prognosis and severity of streptococcal infection, remain poorly understood. *S. pyogenes *has been reported to produce a number of surface-associated and extracellular products contributing to the pathogenesis. In particular, several cell surface proteins have been documented as being involved in adherence and colonization during infection [3].

Many cell surface proteins of gram-positive bacteria share similar structural characteristics that include a variable amino terminus, a central region with repeated sequences, and a cell-associated region with a LPXTGX cell wall anchored motif [4]. A new *S. pyogenes *cell surface protein family, streptococcal collagen-like (Scl) protein, has been identified recently [5-10]. Scl1 (SclA) and Scl2 (SclB), two Scl protein family members, share a similar structure motif, including the LPXTGX motif and a central region composed of variable numbers of Gly-X-X (GXX) collagen-like motifs. Collagen exhibits a triple-helical, elongated protein structure that is the structural component of the extracellular matrix in multicellular organisms. As eukaryotic cells are known to bind to collagen through receptors expressed on cell surfaces [11], it is reasonable to speculate that the Scl protein family may participate in the colonization/binding of *S. pyogenes *to receptors on the host cell. Although the potential role of Scl1 in adhesion has been demonstrated by disrupting the *scl1 *gene in different *S. pyogenes *strains [5,6], the conclusions may be affected by the use of different *S. pyogenes *strains and their carriages of various adhesins. In addition, the presence of other Scl family proteins, as well as other streptococcal surface proteins, which may mask the potential role of Scl1 in adhesion, was not taken into consideration in these studies.

Recent studies have demonstrated that collagen receptor, α2β1 and α11β1 integrins [9,12,13], low density lipoprotein [14], thrombin-activatable fibrinolysis inhibitor [15], cellular fibronectin and laminin [16] and human complement regulatory plasma glycoprotein FH [17] may serve as ligands for Scl proteins. While the *scl1 *gene has been found in all *S. pyogenes *isolates tested, the *scl2 *gene sequence was only detected in some strains [7,10,18]. To determine the *bona fide *nature of Scl1 in colonization and adherence of *S. pyogenes *to human epithelial cells without the potential interference of other streptococcal surface factors, we generated a *scl1 *mutant from a Scl2-defective *S. pyogenes *M29588 strain, and expressed Scl1 in the heterologous bacteria *Escherichia coli*. The adhesion to human epithelial cells was greatly impaired upon the loss of Scl1 in *S. pyogenes *and was markedly increased upon expression of Scl1 on *E. coli*.

## Results

### Identification and analysis of scl1 and scl2 genes in S. pyogenes M29588 strain

**Figure 1:**
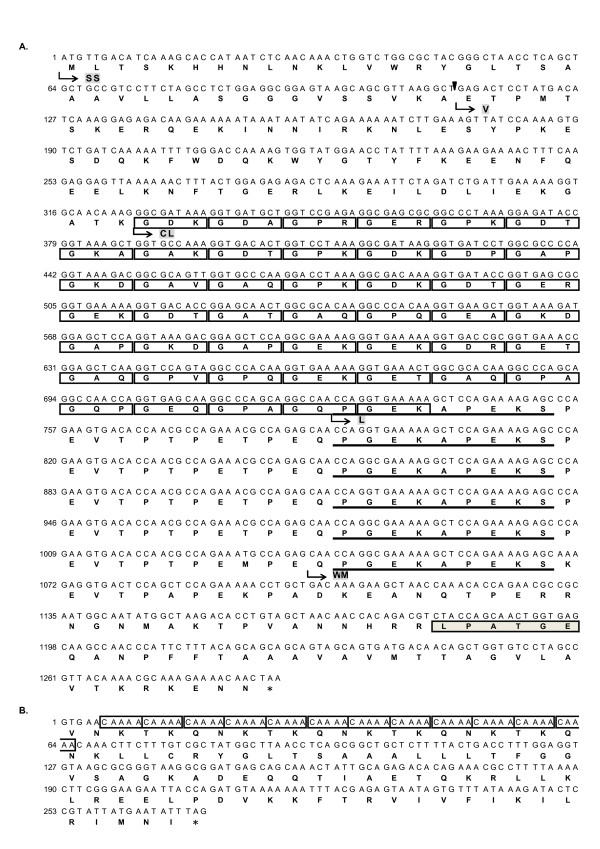
**Nucleotide and inferred amino acid sequences of *scl1 *and *scl2 *genes in *S. pyogenes *M29588 strain (M92 type)**. **(A) ***scl1 *coding sequence consists of 1,287 bp which encodes a protein with 428 amino acids. Scl1 protein is composed of signal sequence (SS) followed by a predicted cleavage site (arrowhead), 71 amino acids in V region, 46 GXX triplet motifs (boxed) in CL region, and 6 PGEKAPEKS repeats (underlined) in L region, and the LPATGE cell wall anchor motif (shaded) in WM region. **(B) **Scl2 protein is translated from the predicted GTG start codon (Val). Thirteen AACAA coding repeats (boxed), located immediately after the GTG start codon, are followed by a premature translation termination at the 89th amino acid residue (asteriated).

To identify genes encoding streptococcal collagen-like surface protein 1 and 2 (*scl1 *and *scl2*) in *S. pyogenes *M29588 strain, full lengths of *scl1 *and *scl2 *genes were amplified by PCR and sequenced. The *scl1 *ORF of *S. pyogenes *M29588 is 1,287 bp, which encodes a protein with 428 amino acid residues (Figure 1A). The Ala38 was the predicted signal peptidase cleavage site. The length of variable (V) region is 71 amino acids. The collagen-like (CL) region is composed of 46 GXX triplet repeats, followed by a gram-positive bacteria cell wall anchor motif (LPATGE) in the cell wall membrane (WM) region. The CL region and cell wall anchor motif are connected by 6 repeats with a PGEKAPEKS core sequence in the linker (L) region.

It has been shown that the expression of Scl2 is controlled by slipped-strand mispairing at sites containing pentanucleotide coding repeats [7,10,18]. In this study, *S. pyogenes *M29588 strain contained the *scl2 *gene, which is predicted to be translated from a putative GTG (Val) start codon (Figure 1B). We identified 13 AACAA pentanucleotide sequence repeats adjacent to the presumed GTG start codon in *S. pyogenes *M29588, followed by a premature translation termination at the 89th amino acid residue upon production of Scl2 protein (Figure 1B). However, the prematurely translated Scl2 protein contains neither CL region nor the anchor motif, suggesting it is not functional and not anchored on the bacteria. These observations show that the *S. pyogenes *M29588 strain appears to express Scl1 protein consisting of 46 GXX triplet repeats and premature non-functional Scl2 protein.

### Loss of adherence to human epithelial cells in S. pyogenes mutant deficient in both Scl1 and Scl2

**Figure 2:**
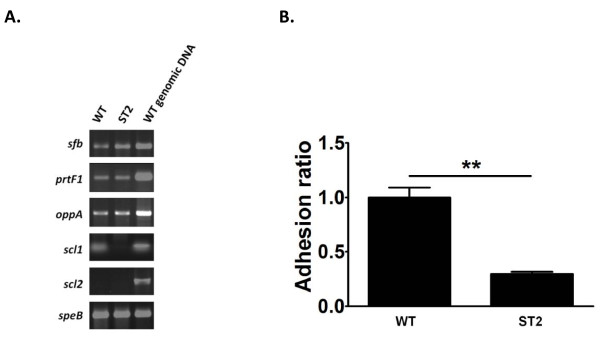
**Expression profile and adhesion ability of *scl1*-mutated *S. pyogenes***. **(A) **mRNA levels in fibronectin binding proteins (*sfb *and *prtF1*), olidopeptidase A (*oppA*), streptococcal collagen-like proteins (*scl1 *and *scl2*), and exotoxin B (*speB*) as an expression control. **(B) **HEp-2 cells were incubated with FITC-conjugated wild-type (WT) and Scl1-mutated *S. pyogenes *(ST2). The adhesion ability is expressed as the ratio of florescence from adherent bacteria to that from inoculated bacteria. Data represent means of five experiments with triplicate samples in each experiment. **, *P *< 0.01 compared with *S. pyogenes *wild-type M29588 strain.

To determine the role of Scl1 in the adherence of *S. pyogenes *to human epithelial cells in the absence of Scl2, we generated a *scl1 *mutant from the Scl2-defective *S. pyogenes *M29588 strain. A kanamycin-resistant mutant (ST2) was identified after electroporation of *S. pyogenes *M29588 with the non-replicating plasmid pPJT8, which contains the internal fragment of the *scl1 *coding region. PCR and Southern blot analysis confirmed the site of mutation, and indicated that the integration occurred through a Campbell-like mechanism (data not shown). No difference in growth rates between the mutant and wild-type strains in TSBY was identified (data not shown), suggesting that the disruption of *scl1 *did not affect major metabolic pathways under a nutrient-enriched condition, and the integration of pPJT8 did not affect the neighboring genes of *scl1*. To further clarify if the mutagenesis strategy affected other surface factors, we determined the expression of fibronectin binding proteins, *sfb *and *prtF1*, and another known adhesin, *oppA*, as well as an exotoxin *speB *as the internal control (Figure 2A). Expression of these four genes was not affected in the *scl1 *mutant ST2. These results suggest that the mutagenesis strategy did not influence other surface factors, and the *scl1 *mutant has not compensated for the loss of this adhesin by altering expression profiles for other potential surface binding proteins we tested. In addition, DNA sequence and the number of pentanucleotide repeats of *scl2 *were not altered in ST2 (data not shown).

To further investigate the role of Scl1 in mediating the adherence of *S. pyogenes *to human epithelial cells, wild-type and *scl1-*mutated *S. pyogenes *ST2, in the exponential phase, were examined for adhesion to human HEp-2 epithelial cells. Adhesion of ST2, was decreased about 70% compared with that of the wild-type (*P *< 0.01, Figure 2B), suggesting that Scl1 is critical in the adherence of *S. pyogenes *to human epithelial cells.

### Ectopic expression of Scl1 on *E. coli*

**Figure 3:**
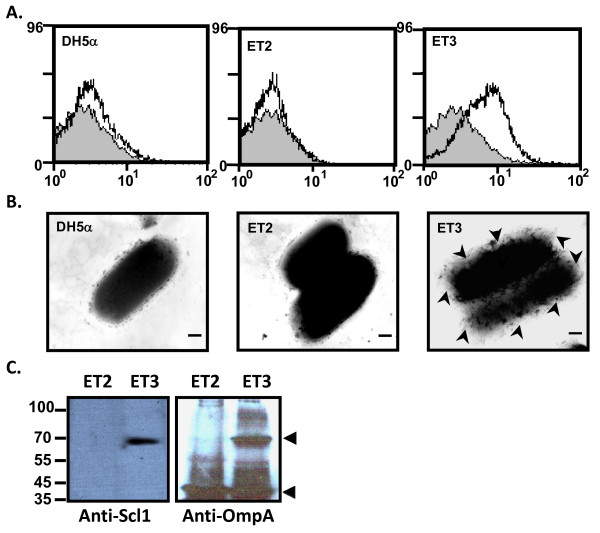
**Ectopic expression of Scl1 on *E. coli***. **(A) **FACS analysis on whole bacteria pre-incubated with (white profile) or without (gray profile) anti-Scl1 antibodies, followed by FITC-conjugated secondary antibodies. **(B) **Electron microscope view of whole bacteria after negative staining with sodium phosphotungstate. Asterisks indicate ectopic expressed Scl1 on the *E. coli *surface. Bars represent 100 nm. ET2, *E. coli *expressing vector only. ET3, *E. coli *expressing Scl1. **(C) **Western blot analysis with anti-Scl1 (left panel) and anti-OmpA (right panel) antibodies in the outer membrane fraction of ET2 and ET3.

To exclude the interference of other streptococcal surface factors during the adhesion, and to test whether Scl1 is sufficient to mediate the adherence to human epithelium cells, we expressed Scl1 on the heterologous bacteria *E. coli*. Signal sequence (SS), WM region, and part of the L region of Scl1 were not constructed into OmpA-containing vector. *E. coli *DH5α with OmpA-containing vector was represented as ET2, whereas *E. coli *DH5α with truncated Scl1-OmpA construct was represented as ET3. To confirm the expression of Scl1 protein on the surface of *E. coli*, we performed FACS analysis on whole bacteria. A right-shift of peak fluorescence recognized by anti-Scl1 antibodies was observed in ET3, but not in either *E. coli *DH5α or ET2. (Figure 3A). Consistent with this observation, the negative staining of electron microscopy revealed hairy structures in ET3, but these structures were not identified in either *E. coli *DH5α or ET2 (Figure 3B). To further demonstrate that Scl1 was ectopically expressed on *E. coli*, outer membrane fraction of proteins was isolated from ET2 and ET3. Western blot analysis with anti-Scl1 antibodies identified Scl1 in the outer membrane fraction of ET3 but not in that of ET2 (Left panel, Figure 3C). Consistently, a molecular weight shift was revealed by anti-OmpA antibodies in the outer membrane fraction of ET3 (Right panel, Figure 3C). Thus, our data confirmed that Scl1 protein was ectopically expressed on *E. coli *and can be detected by anti-Scl1 antibodies.

### Adherence of Scl1-expressed E. coli to human epithelial cells

**Figure 4:**
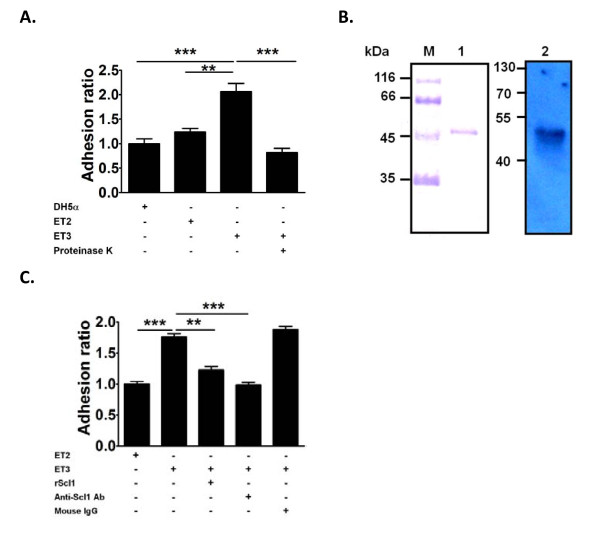
**Adhesion abilities of *E. coli *to HEp-2 cells**. **(A) **Adhesion of FITC-conjugated ET2, and ET3 to HEp-2 cells. The adhesion ability is expressed as the ratio of florescence from adherent bacteria to that from inoculated bacteria. Bacteria were treated with proteinase K before FITC conjugation. Data represent means of five experiments with triplicate samples in each experiment. ET2, *E. coli *expressing vector only. ET3, *E. coli *expressing Scl1. **(B) **SDS-PAGE and western blot analysis of purified recombinant Scl1 protein. Lane 1 indicates the SDS-PAGE of purified rScl1. Lane 2 indicates the purified rScl1 protein confirmed by western blot analysis using anti-Scl1 antibody. rScl1 is indicated by a 48 kDa band. **(C) **Inhibition of binding by rScl1 protein and anti-Scl1 antibody. Prior to the adhesion assay, HEp-2 cells were pre-treated with rScl1 protein and ET3 were pre-treated with anti-Scl1 antibody and mouse IgG, respectively. **, *P *< 0.01 and ***, *P *< 0.001.

Adhesion analysis demonstrated that Scl1-expressed *E. coli *ET3 dramatically increased its adherence to HEp-2, compared with that of vector-expressed *E. coli *ET2 and *E. coli *DH5α (Figure 4A). Pre-incubation of *E. coli *ET3 with proteinase K significantly attenuated the Scl1-mediated increase in adhesion, suggesting that Scl1 proteins on *E. coli *are critical for this binding. Thus, the addition of Scl1 on the surface of the heterologous bacteria greatly enhances their adherence to human epithelial cells.

To directly address the role of Scl1 in the binding process, we performed competition studies using anti-Scl1 antibodies and recombinant Scl1 (rScl1) protein. Polyclonal anti-Scl1 antibodies were generated in 4-week-old BALB/c mice. The full-length rScl1 protein containing sequences shown in Figure 1A was generated and confirmed by SDS-PAGE as a single band of approximately 48 kDa (Lane 1, Figure 4B) and by western blot analysis with anti-Scl1 antibodies (Lane 2, Figure 4B). Both pre-incubation of HEp-2 cells with rScl1 and pre-incubation of ET3 bacteria with anti-Scl1 antibodies significantly blocked the adherence of *E. coli *ET3 to human epithelial cells (Figure 4C). The adherence of *E. coli *ET3 to HEp-2 cells was not affected by pre-incubation of ET3 bacteria with non-specific mouse IgG. These results reveal both the importance and sufficiency of Scl1 in mediating the adherence of bacteria to human epithelial cells.

### Adherence through protein receptor(s) on epithelial cells

**Figure 5:**
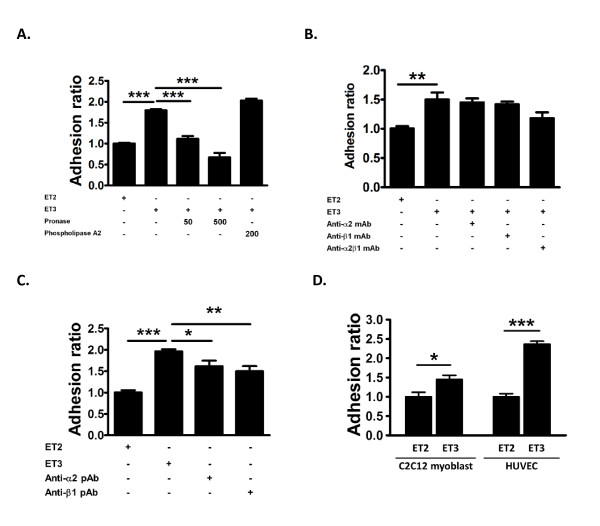
**Adherence through protein receptors on HEp-2 cells**. **(A) **Adhesion of *E. coli *to surface-modified HEp-2 cells. HEp-2 cells were pre-incubated with medium alone, pronase (50 and 500 μg/ml), and phospholipase A2 (200 μg/ml), respectively, prior to the adhesion assay. **(B) **Adhesion of *E. coli *to HEp-2 cells with pre-treatment of monoclonal antibodies (mAb) against α2, β1, and α2β1 integrins. **(C) **Adhesion of *E. coli *to HEp-2 cells with pre-treatment of polyclonal antibodies (pAb) against α2 and β1 integrins. **(D) **Adhesion of *E. coli *to C2C12 myoblasts and HUVECs. Data represent means of five experiments with triplicate samples in each experiment. *P < 0.05, **P < 0.01, and ***P < 0.001.

Our previous data showed that the adhesion was affected when Scl1-expressed *E. coli *was pre-incubated with proteinase K, suggesting that the adhesion is mediated through a protein-like molecule on the bacteria. To further determine the corresponding side of surface molecules on epithelial cells mediating this binding process, HEp-2 cells were treated with pronase and phospholipase A2 to modify the protein and lipid contents on the cell membrane, respectively [19]. Treatment of pronase significantly inhibited the binding of ET3 to epithelial cells in a dose-dependent manner (Figure 5A). In contrast, treatment of phospholipase A2 did not affect the binding of ET3 to epithelial cells (Figure 5A). These results suggest that a protein receptor for Scl1 on epithelial cells is likely to mediate this binding event.

It has been proposed that α2β1 and α11β1 integrins might serve as receptors in mediating the Scl1 adherence to epithelial cells [9,12,13]. To determine the role of integrins in the Scl1-mediated binding process, we used monoclonal antibodies against α2, β1, and α2β1 integrins, and performed a competition assay. Pretreatment of monoclonal antibodies against α2, β1, and α2β1 integrins to HEp-2 cells did not affect Scl1-mediated increase in the adhesion of *E. coli *to human epithelial cells (Figure 5B). However, we observed a trend, although not significant, toward reduction in the adhesion of *E. coli *to HEp-2 cells in the presence of monoclonal α2β1 antibodies, suggesting that α2β1 integrin is involved to some extent in the Scl1-mediated binding process. To avoid the lack of interference of the abovementioned monoclonal antibodies in the binding interaction, we employed polyclonal antibodies against α2 and β1 integrins. Polyclonal antibodies against α2 and β1 integrins significantly decreased Scl1-mediated adhesion of *E. coli *to human epithelial cells (Figure 5C). These results suggest that protein receptors α2 and β1 integrins underlie the Scl1-dependent binding to human epithelial cells.

To further examine the Scl1-mediated adhesion of *E. coli *to other eukaryotic cell types known for expression of collagen receptors, we employed two types of cell lines, C2C12 myoblast and human umbilical vein endothelial cell (HUVEC) for the adhesion assay. C2C12 cells are known to express β1 integrins [20], whereas primary HUVECs express α2β1 integrins [21]. Our results show that Scl1-expressed *E. coli *ET3 exhibited significantly increased adherence to both C2C12 and HUVEC cells, compared to control ET2 (Figure 5D). Thus multiple eukaryotic cell types may bind and adhere to Scl1-expressed *E. coli*.

## Discussion

The Scl1 protein in the *S. pyogenes *M29588 strain (M92 type) contains a predicted signal peptidase cleavage site on Ala38, 71 amino acids in V region, 46 GXX repeats in CL region, 6 conserved repeats (PGEKAPEKS) in L region, and followed by a cell wall anchor motif (LPATGE). It has been proposed that the V-region primary sequence in Scl1 is M type associated [7]. Based on the previous study in characterization of the *scl1 *gene among 21 different M type strains [6], the length of V region in M92 strain is identical to those in M49 and M56 strains. The number of GXX repeats in CL region in M92 strain is equal to those in M2 and M49 strains. The number of PGEKAPEKS repeats in L region in M92 strain is the same with those in M4 and M9 strains. These findings demonstrate significant and extensive genetic variations among clinical isolates of *S. pyogenes*.

Rasmussen et al. demonstrated that an isogenic Scl1-deficient M1 strain (AP1) with 57 GXX repeats did not alter its adhesion ability to Detroit 562 pharyngeal cells [5]. In contrast, Lukomski et al. demonstrated that two independent isogenic Scl1-deficient M1 strains (MGAS 6708 and 5005) with 50 GXX repeats had significantly reduced adherence to human A549 epithelial cells [6]. Although the differences on the surface of various host epithelial cells cannot be excluded, this inconsistency may stem from the carriage of various group A streptococcal adhesins and potential interference of another Scl family member, Scl2. The role of Scl2 in adhesion has been directly addressed in another study by Rasmussen et al. showing that Scl2-deficient isogenic mutants had decreased adherence to human fibroblast cells, but no influence on adherence to pharyngeal cells [18]. Thus, Scl2 appears to be involved in the adhesion process, and the presence of Scl2 could therefore potentially influence and mask the effect of Scl1 in the adhesion. However, Scl2 production in all M1-type strains investigated so far is early terminated at the level of translation [7,18]. In our study, we also demonstrated that the *S. pyogenes *M29588 strain expresses a pre-terminated Scl2, which contains neither CL region nor anchor motif, according to our sequence analysis. These findings suggest that Scl2 in this particular strain is not functional due to the absence of CL region, and is not anchored on the cell membrane because of the lack of an anchor motif. Our adherence results based on this Scl2-defective *S. pyogenes *M29588 strain provide evidence for the contribution of Scl1 on the binding to host epithelial cells.

While Rasmussen et al. used a Scl2-defective AP1 strain to demonstrate that Scl1 mutation does not affect adherence of bacteria to pharyngeal cells [5], their study may have utilized a background where the Scl1 mutation was compensated for by other adhesins, such as protein H [22], C5a peptidase [23]. In our study, we also identified the expression of some surface proteins in this M29588 strain. To exclude the interference of other streptococcal surface factors during Scl1-mediated adhesion, the heterologous expression of Scl1 on *E. coli *would be an alternative. The outer membrane of Gram-negative bacteria presents an effective barrier that restricts the release of proteins from the bacteria [24]. Many peptides have been inserted within external loops of various outer membrane proteins and have been shown to be exposed on the surface of intact *E. coli *by immunochemical techniques [24-26]. In addition, studies have demonstrated that OmpA chimeric proteins were stably anchored on the external side of the bacteria [24,25,27]. Here we demonstrated that truncated Scl1 fused with OmpA was directed to the outer membrane fraction of *E. coli *by western blot analysis, and likely exposed on the surface of *E. coli *by FACS analysis. While ectopic expression of Scl1 on the heterologous bacteria *E. coli *is an alternative approach to reduce the potential interference of other factors on the surface of *S. pyogenes*, there are some limitations in our study. For example, it can not be ruled out that Scl1 protein was secreted to the periplasmic space, because Scl1 was constructed after the OmpA signal sequence. To avoid this problem, we performed FACS analysis on whole bacteria using Scl1 antibodies to detect the location of Scl1 in/on *E. coli*. FACS analysis has been widely used in identification of cell surface molecules in many immunologic and hematologic studies. Furthermore, we isolated proteins from the outer membrane fraction and confirmed the existence of Scl1 by western blot analysis with antibodies against Scl1 and its fusion protein OmpA. However, the proper folding of ectopically expressed Scl1 and the integrity of the outer membrane of *E. coli *account for other issues influencing our interpretation of Scl1 in adhesion. Nevertheless, our findings concerning the adherence of Scl1-expressed *E. coli *to human epithelial cells unequivocally show that Scl1 contributes significantly to the adhesion of bacteria to human epithelial cells.

Collagen is a triple-helical, elongated protein structure that is the main structural component of the extra-cellular matrix in all multicellular organisms. Collagen-like sequences are found not only in proteins of multicellular organisms but also in proteins of microorganisms, such as a pullulanase in *Klebsiella pneuminiae *[28] and a platelet aggregation-associated protein in *S. sanguis *[29,30]. Moreover, collagens interact with several macromolecules in a specific manner, suggesting that the collagen-like repeat sequences not only play a basic structural role, but also have a functional significance. Many eukaryotic cells bind collagen through integrins expressed on their surface [11]. Studies have demonstrated that the recombinant Scl1.41 protein interacted with α2β1 and α11β1 integrins, induced intracellular signaling in host cells, and promoted the internalization of *S. pyogenes *[9,12,13]. While the hypothesized region mediating the binding to α2β1 and α11β1 integrins in the recombinant Scl1.41 is in a motif called the GLPGER motif [9,12,13], Scl1 protein of *S. pyogenes *M29588 strain in our study does not contain the GLPGER motif. The novel aspect of this study is the observation that, in this Scl1 sequence type, the GLPGER motif is absent, yet adherence is maintained. Nevertheless, our results indicate that protein receptors, α2 and β1 integrins, contribute to Scl1-dependent binding to the surface of human epithelial cells. Consistently, Scl1-mediated adhesion was also demonstrated in other eukaryotic cell types known for expression of collagen receptors.

## Conclusions

In summary, we demonstrated that loss of Scl1 in a Scl2-defective *S. pyogenes *strain decreased the adhesion of bacteria to human epithelial cells. Ectopic expression of Scl1 in the heterologous Gram-negative bacteria *E. coli *promoted the adhesion of bacteria to epithelial cells. The increase in adhesion was nullified by proteinase K, rScl1 protein and anti-Scl1 antibody. This binding event appears to be mediated through protein receptors, α2 and β1 integrins, instead of a lipid component, on the surface of epithelial cells. Our results underscore the importance of Scl1 in the adherence of *S. pyogenes *to human epithelial cells. Understanding the mechanisms by which *S. pyogenes *adheres to nasal epithelial cells may lead to alternative therapeutic methods of decolonization and decrease the dependence on antibiotics.

## Methods

### Bacterial strains and plasmids

*S. pyogenes *strain M29588 (*emm *sequence type 92) was recovered from a patient with necrotizing fasciitis at the Tzu-Chi General Hospital. *S. pyogenes *cultures were grown in tryptic soy broth supplemented with 0.5% yeast extract (TSBY). *E. coli *DH5α was grown in Luria broth (LB). Plasmid pSF151 was kindly provided by Dr. Tao of the University of Missouri, Kansas City, USA [31]. Plasmid pST1, which contains the truncated OmpA fusion protein derived from pCR2.1-TOPO (Invitrogen), was kindly provided by Dr. C. Y. Chen of National Taiwan University, Taipei, Taiwan. ET2 and ET3 are *E. coli *DH5a containing plasmids pST1 and pPJT9, respectively. *E. coli *was transformed according to the method of Sambrook et al. [32]. *S. pyogenes *was electroporated according to the method of Schalen et al. [31].

### Cloning of scl1 and scl2

The internal *scl1 *gene was amplified by PCR using *S. pyogenes *M29588 DNA as a template with the primers of *scl1*-4 (5'-AACTGCAGCCTTTTTCACCCTTTTCGCC-3') and *scl1*-5 (5'-GGGGTACCTTTGGAGGCGGGGCAAGCA-3'), while the full-length *scl1 *gene was amplified by primers of *scl1*-6 (5'-TCCCCCGGGATGTTGACATCAAAGCAC-3') and *scl1*-7 (5'-TCCCCCGGGTTAGTTGTTTTCTTTGCG-3') based on the previously published sequence [6]. Primers of *scl2*-3 (5'-GTGAACAAAACAAAA-3') and *scl2*-4 (5'-TTAGTTGTTTTCTTG-3'), obtained from the Streptococcal Genome Sequencing database, were used to amplify the *scl2 *gene. The underlined sequences represent the restriction sites. After amplification, the 0.5-kb internal *scl1 *PCR product was digested with *Kpn*I and *Pst*I, and inserted into plasmid pSF151 to generate plasmid pPJT8. Truncated Scl1 from V region to part of L region was amplified by primers of *scl1*-8 (5'-TCCCCCGGGGAGACTCCTATGACATCA-3') and *scl1*-2 (5'-TCCCCCGGGTTTGGTTAGCTTCTTTGTC-3'), digested with *Sma*I, and inserted into OmpA-containing vector pST1 to generate plasmid pPJT9. The construction was analyzed by endonuclease digestion and DNA sequencing (ABI-3730 auto-sequencer, Applied Biosystems). The 1.5-kb fragment of *scl2 *gene was analyzed directly by DNA sequencing. The *scl1 *and *scl2 *sequences reported here were deposited in GenBank under accession numbers DQ166850 and DQ166851.

### Bacterial RNA extraction and RT-PCR

Extraction of total RNA was done as described previously with slight modification [33]. Briefly, bacteria were harvested, washed, resuspended with buffer containing lysozyme and mutanolysin, and incubated to weaken cell walls. Bacterial pellets were collected and resuspended. Extraction of RNA was done by mixing with hot phenol followed by vortex and centrifugation. The upper aqueous phase was collected and precipitated. RNA was treated with DNase and re-extracted again. 1 μg extracted RNA was reverse-transcribed to cDNA in total 20 μl reactive solution by Improm II RT kit (Promega). The expression of *sfb*, *prtF1*, *oppA*, *speB*, *scl1*, and *scl2 *was assessed by PCR with primers *sfb*-1 (CCTCTAGCGGGTGAGTCT), *sfb*-2 (AATGGAACACTGAATTCGGACGGG), *prtF1*-1 (TTTTCAGGAAATATGGTTGAGACA), *prtF1*-2 (TCGCCGTTTCACTGAAACCACTCA), *oppA*-1 (TGGTATACGGCTGATGGTGA), *oppA*-2 (GCTTTCTTACCGGCATCTTG), *speB*-1 (TGATGGCTGATGTTGGTATTTC), *speB*-2 (ATTCTTTGTCAATTTGTGCTTCC), *scl1*-6 (ATGTTGACATCAAAGCAC), *scl1*-4 (CCTTTTTCACCCTTTTCGCC), *scl2*-1 (TGCTGACCTTTGGAGGTGC), and *scl2*-2 (CGCCTGTTGCTGGCAATTGTC). Genomic DNA was used as a positive control to confirm the size of PCR product, and the extracted RNA was used as a negative control to exclude the possibility of DNA contamination.

### Adhesion assay

Human epidermoid carcinoma epithelial cells (HEp-2; ATCC CCL-23) and C2C12 mouse myoblasts (ATCC CRL-1772) were cultured in DMEM supplemented with 10% FCS. HUVECs were cultured on 0.04% gelatin-coated (Sigma) plates in M199 supplemented with 2 mM L-glutamine (Invitrogen), 10% FBS, and 25% EGM. Adhesion of FITC-conjugated bacteria to cells was measured using a previously described method with slight modifications [34,35]. Bacteria were suspended in cell culture medium to a density of 4 × 10^8 ^cells/ml. FITC-conjugated bacterial suspension was added to the confluence cells at a M.O.I. of 100 and incubated for 2 hrs at 37°C. The fluorescence of each well was measured by a CytoFluor II flourescence reader (Millipore) with excitation and detection wavelength of 485 nm and 530 nm, respectively. Compared to the results from the conventional plating experiment, the FITC conjugation did not affect the adherence of bacteria.

### Blocking assay

For the proteolytic treatment of bacteria, the bacterial suspension (10^8 ^CFU/ml) was incubated with proteinase K (10 μg/ml) for 1 h at 37°C. The suspension was washed and re-suspended in 1 ml of PBS for the subsequent FITC-conjugation and adhesion assay. In the antibody blocking assay, FITC-conjugated bacteria was incubated with anti-Scl1 antibody (10 μg/ml) for 30 min at room temperature. In the recombinant protein blocking assay, HEp-2 cells were pre-incubated with recombinant Scl1 protein (10 μg/ml), and subsequently incubated with FITC-conjugated bacteria for the adhesion assay. To enzymatically modify cell surface proteins, confluence HEp-2 cells were incubated with pronase (500 and 50 μg/ml; Sigma) or phospholipase A2 (200 μg/ml; Sigma) in FCS-free DMEM for 30 min at 37°C, washed twice with PBS, and then incubated with FITC-conjugated bacteria for the adhesion assay. For the integrin blocking assay, confluence HEp-2 cells were incubated with antibodies (10 μg/ml) against α2 (P1E6, monoclonal, Chemicon International; P17301, polyclonal, Millipore), β1 (P4G1, monoclonal, Chemicon International; P05556, polyclonal, Millipore), α2β1 (BHA2.1, monoclonal, Chemicon International) integrins and mouse IgG (Sigma) for 30 min before the incubation with FITC-conjugated bacteria for the adhesion assay.

### Electron microscopy

Drops of bacterial suspension fixed with 2.5% glutaraldehyde were concentrated and placed on formvar-coated copper grids for 1 min. After removal of excess fluid by placing on filter paper, the wet residues were immediately covered with the stain for 30 sec. The grid was air-dried before examination for negative staining electron microscopy.

### FACS analysis

Surface-detection of Scl1 in *E. coli *was performed by FACS analysis. Approximately 1 × 10^7 ^bacteria were incubated with mouse anti-Scl1 antibody (1:1000) for 1 hr and subsequently with FITC-conjugated goat anti-mouse IgG (1:1000, Amersham Biosciences) for 30 min. The fluorescence of adhered bacteria was analyzed by a FACS-Scan flow cytometer (Beckton-Dickinson).

### Surface protein isolation

Outer membrane proteins were isolated from bacteria cultures according to a protocol by Fountoulakis and Gasser [36]. Briefly, the overnight *E. coli *culture was pelleted and the bacteria were resuspended. After shacking and a centrifugation, the new pellet was resuspended and disrupted 3 times by sonication. To remove unbroken cells and debris, sonicated bacteria were centrifuged at 3,000 rpm and subsequently the supernatants were centrifuged at 90,000 rpm. To solubilize the inner membrane protein, the pellet was incubated with 2 ml 2% sodium N-laung sarcosinate and subsequently the supernatants were centrifuged at 90,000 rpm. The pelleted outer membrane proteins were resuspended. OmpA expression pattern performed by western blot using anti-OmpA antibody was represented as an internal control.

### Recombinant protein and preparation of antibody

The 1.3-kb full-length *sc1l *gene was cloned into plasmid pQE30 to construct plasmid pPJ10. The recombinant protein was expressed after isopropyl-β-D-thiogalactopyranoside induction. The expressed protein containing the His_6 _tag was separated in a Ni-chelated column (Amersham Biosciences) and eluted by a 0 to 50 mM imidazole gradient. The purified protein was verified by SDS-PAGE and western blot analysis with anti-His monoclonal antibody (Invitrogen). Antibody against purified rScl1 was raised in 4-week-old BALB/c mice. One hundred microgram of rScl1 was applied in the initial immunization of BALB/c mice, with succeeding injections 2 and 4 wks thereafter. Anti-Scl1 mouse IgGs were enriched and purified by affinity chromatography with a column made of Sepharose conjugated to protein A.

### Data analysis

Values were reported as mean ± SEM. Statistical analysis was conducted using JMP software (SAS Institute). Student's *t*-test was used for comparisons between groups and differences were considered to be statistically significant with *P *value less than 0.05.

## Authors' contributions

SMC, YST, and PJT designed the study and wrote the paper. SKL helped draft the manuscript. LCW, CSC and YHL participated in strain construction, RT-PCR, protein purification, antibody generation, cell adhesion assays and FACS analysis. CMW carried out the electron microscopy. All authors read and approved the final manuscript.
